# Particulate matter exposure induces maternal scalp hair loss after birth in C57/B6 mouse via alteration of inflammatory and apoptotic pathways

**DOI:** 10.3389/fendo.2026.1766198

**Published:** 2026-05-08

**Authors:** Gee Soo Jung, Min Jung Lee, Wooseok Im, Hyemin Park, Inha Lee, Jae Hoon Lee, Hyeno Ho Ku, Sang Eun Lee, SiHyun Cho, Young Sik Choi

**Affiliations:** 1 Department of Integrative Medicine, Yonsei University College of Medicine, Seoul, Republic of Korea; 2 Department of Obstetrics and Gynecology, Gangnam Severance Hospital, Yonsei University College of Medicine, Seoul, Republic of Korea; 3 Institute of Women’s Life Medical Science, Yonsei University College of Medicine, Seoul, Republic of Korea; 4 Department of Medical Device Engineering and Management, Yonsei University College of Medicine, Seoul, Republic of Korea; 5 Department of Dermatology, Gangnam Severance Hospital, Yonsei University College of Medicine, Seoul, Republic of Korea; 6 Department of Obstetrics and Gynecology, Division of Reproductive Endocrinology Severance Hospital, Yonsei University College of Medicine, Seoul, Republic of Korea

**Keywords:** apoptosis, fibrosis, inflammation, particulate matter, postpartum hair loss

## Abstract

PM 2.5 exposure is associated with a variety of health effects, including effects on the reproductive and skin. However, the relationship between PM2.5 exposure and postpartum hair loss has not been investigated. In this study, we evaluated the effect of PM2.5 exposure on hair loss after birth in mouse model and analyzed possible associated molecular changes. Female mice were exposed to PM2.5 using nasal inhalation method. After 4 weeks, mating tests were conducted and postpartum scalp tissues from PM2.5-exposed mice and those without exposure were harvested and analyzed. Then, human immortalized keratinocyte cell line (HaCaT cells) and fibroblasts were cultured and treated with PM2.5 for 24 hours. Changes in the inflammatory, apoptotic, fibrotic, and proliferative pathways were evaluated. Postpartum scalp hair loss was evident in PM2.5 exposed mice group with significant morphological changes in scalp tissues. The expression levels of IL-6, IL-1β, TNF-α and p-NF-κB, Caspase-3, the BAX/Bcl-2 ratio, COL1A1, MMP2 and MMP9 were significantly higher in the PM2.5-exposed group than in the control group. The expressions of were elevated in PM2.5 exposed group than the controls, where the expressions of PR-B, PR-A, CD34 and K15 were significantly lower in the exposed group. Histologic analysis showed that PM2.5 exposed postpartum scalp showed thickened stratum corneum, migration of hair follicles deeper into the dermis with a decrease in the number of hair follicles. Increased collagen density in the dermis was also observed in scalp tissues from the PM2.5-exposed group. *In vitro* experiments showed that PM2.5 exposure significantly increased expressions of p-NF-κB/NF-κB, p-c-jun/c-jun, p-p53/p53, p27, Caspase-3 and BAX/Bcl-2, where p-ERK/ERK and VEGF expressions were significantly reduced in HaCaT cells and fibroblasts. These findings suggest that PM2.5 exposure induces postpartum hair loss via alterations of inflammatory and apoptotic pathways. PM2.5 exposure induces significant downregulation of progesterone receptors and reduces the hair follicle stem cells (HFSCs) population, which may contribute to the exacerbation of postpartum hair loss.

## 1 Introduction

Air pollution consists of various harmful substances including Particulate matter (PM), industrial gases, heavy metals, and organic chemicals, all of which are increasingly recognized as significant threats to human health (1, 2). PM, generated from traffic emissions and industrial facilities, is classified as PM10 or PM2.5 depending on its aerodynamic diameter (3). Fine particles like PM2.5 are small enough to enter deep into the lungs, potentially affecting multiple organs by triggering inflammation and oxidative damage (4, 5). Exposure to PM2.5 has been shown to have acute and chronic negative effects on various systems, including reproductive and skin systems (6–8). In the dermal system, it is important to understand how exposure to PM2.5 can affect scalp function and contribute to postpartum hair loss, especially during hormonally vulnerable periods such as the postpartum period.

Due to its small size, PM2.5 penetrates the skin barrier and accumulates systemically, leading to the production of reactive oxygen species (ROS) production and inflammatory responses (9–11). These processes disrupt the normal environment of the hair follicles and can interfere with the hair growth cycle (12). In particular, exposure to PM2.5 has been linked to increased cell death in hair follicle keratinocytes and reduced activity of dermal papilla (DP) cells, which are important for maintaining healthy hair growth (10, 13, 14). Harmful substances in PM2.5, such as heavy metals and polycyclic aromatic hydrocarbons (PAHs), induce oxidative stress and inflammation, damaging hair follicles and potentially contributing to hair loss (12, 15, 16). However, to our knowledge, there are no epidemiological studies directly linking ambient PM2.5 exposure with postpartum hair loss, highlighting a gap in understanding environmental contributors to postpartum scalp vulnerability.

Hair follicle cycling includes anagen (growth), catagen (regression), and telogen (resting) phases, which are regulated by specific molecular markers (17). For example, β-catenin is associated with anagen, p53 with catagen, and stem cell markers such as CD34 and K15 contribute to hair follicle maintenance (18–20). The postpartum period is characterized by rapid hormonal fluctuations, notably the sharp decline of circulating estrogen and progesterone levels after birth (21, 22). These hormonal changes are closely associated with telogen effluvium, a temporary hair shedding condition caused by premature transition of hair follicles into the telogen phase (23, 24). While hormonal changes are considered the main cause of postpartum hair loss, external environmental factors, including PM2.5 exposure, may further aggravate scalp susceptibility (13, 25, 26). PM2.5 has been reported to induce oxidative stress, inflammation, and apoptotic signaling in skin cells (27, 28), but its role in postpartum hair loss remains unexplored. Although mice and humans share similar hair follicle cycling phases, mice exhibit a more synchronized hair cycle, whereas humans display an asynchronous pattern, which should be considered when interpreting translational relevance (29).

The aim of this study was to investigate the potential mechanisms by which PM2.5 exposure affects postpartum scalp hair loss. Using a mouse model and complementary *in vitro* experiments with human immortalized keratinocyte cell line (HaCaT cells) and fibroblasts, we examined the effects of PM2.5 on inflammatory and apoptotic pathways. This study provides insight into how environmental PM2.5 exposure may exacerbate postpartum hair loss. To our knowledge, this is the first study to show that PM2.5 exposure is associated with alterations in progesterone receptor expression and hair follicle stem cell-related markers in the context of postpartum hair loss using both *in vivo* and *in vitro* models.

## 2 Materials and methods

### 2.1 Mouse model and PM exposure

**Figure 1:**
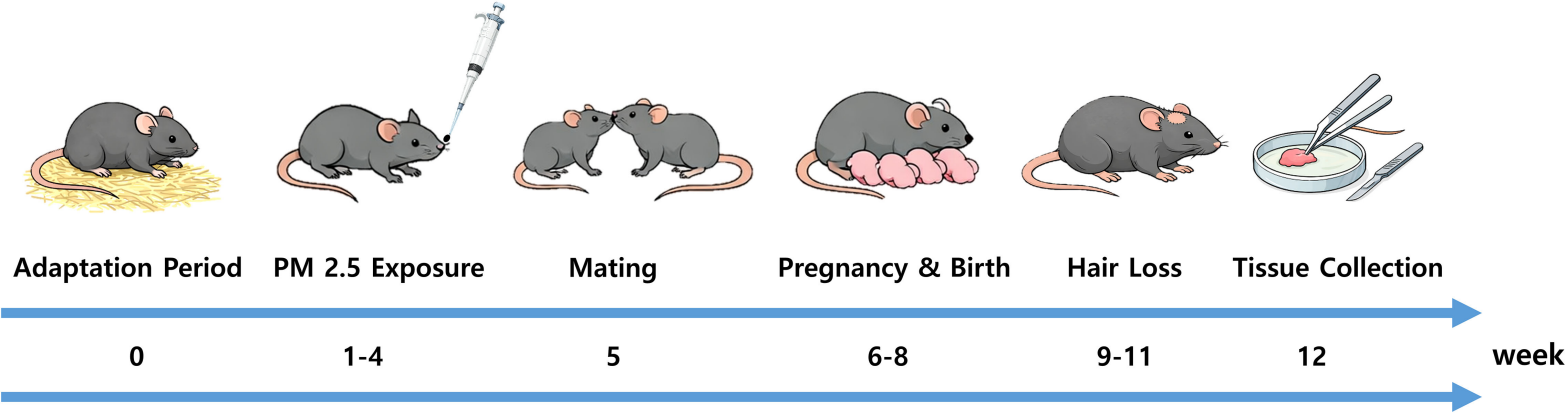
Schematic illustration of the experimental design. After a 1-week acclimation period (Week 0), mice were exposed to PM2.5 for 4 weeks (Weeks 1–4), followed by a 1-week mating period (Week 5). Pregnancy and delivery occurred over the subsequent 3 weeks (Weeks 6–8). Postpartum hair loss was observed during Weeks 9–11, and scalp tissues were collected at Week 12.

All experimental procedures followed the guidelines outlined in the National Institutes of Health (NIH) Guide for the Care and Use of Laboratory Animals and were approved by the Institutional Animal Care and Use Committee (IACUC) of Gangnam Severance Hospital, Yonsei University College of Medicine, Seoul, Republic of Korea (Protocol No. 2021-0284). Male and female C57BL/6 mice were obtained from Orient Bio Inc. (Seongnam, Republic of Korea) and maintained under a 12:12-hour light/dark cycle at 21 °C. Eight-week-old female mice were randomly assigned to either a vehicle control group (DMSO) or a PM2.5-exposed group (PM2.5, 20 mg/kg/day; NIST^®^ SRM^®^ 1648a; Sigma-Aldrich, St. Louis, MO, USA). PM2.5 (NIST^®^ SRM^®^ 1648a; Sigma-Aldrich) is an urban particulate matter reference material containing a mixture of organic compounds, polycyclic aromatic hydrocarbons (PAHs), inorganic elements, and trace metals such as Fe, Pb, and Zn, as specified in the certificate of analysis. Mice were exposed to PM2.5 via intranasal administration five times per week for four weeks. The PM2.5 dose was selected based on previous studies using mouse models (30, 31). While this dose is higher than typical environmental exposure in humans, it enables mechanistic assessment within a practical experimental timeframe. DMSO at 0.1% was used as a vehicle control based on previous reports demonstrating that this concentration does not induce local or systemic inflammatory responses and is widely used for intranasal delivery in experimental models (32, 33). Although PM2.5 was delivered via intranasal instillation, it has been reported to translocate from the respiratory tract into systemic circulation and accumulate in peripheral tissues, including the skin (34). Therefore, intranasally administered PM2.5 is expected to reach the scalp via systemic distribution, mimicking the systemic effects of environmental PM2.5 exposure. Following the exposure period, female mice were paired with males (two groups of three females and one group of two females, each with one male) for one week, and mating success was confirmed by detection of vaginal plugs. To assess the combined effects of PM2.5 exposure and reproductive status, animals were categorized into four groups: non-pregnant control, non-pregnant PM2.5-exposed, postpartum control, and postpartum PM2.5-exposed mice. Scalp tissues were collected after parturition and used for subsequent histological and molecular analyses, with eight mice per group. The overall experimental design is illustrated in **Figure 1**.

### 2.2 Culture of HaCaT cells and fibroblasts

HaCaT cells and human dermal fibroblasts were obtained from the Department of Dermatology, Gangnam Severance Hospital. Each samples were minced into small fragments and digested in PBS containing 2.0 mg/mL collagenase type I (Gibco, Waltham, MA, USA) at 37 °C for 2 h with 5% CO_2_. The digested material was filtered through a 40 μm strainer (BD Biosciences, San Jose, CA, USA) to remove debris. Cells were cultured in Dulbecco’s modified Eagle’s medium (DMEM; Cytiva, Marlborough, MA, USA) supplemented with 10% fetal bovine serum (FBS; Gibco) and 2% penicillin–streptomycin (P/S; Cytiva, Marlborough, MA, USA). Cultures were maintained at 37 °C in a humidified incubator with 5% CO_2_, and cells at passages 3–5 were used for experiments. DP cells are central regulators of hair-follicle growth. However, due to the limited availability of human scalp DP cells, HaCaT cells and fibroblasts were used as alternative *in vitro* models (35).

### 2.3 PM preparation and *in vitro* exposure

PM2.5 was dissolved in dimethyl sulfoxide (DMSO; Sigma-Aldrich) with ultrasonic agitation. The composition of this standard PM2.5 is provided by the manufacturer’s Certificate of Analysis, which details the elemental and organic constituents. For *in vitro* experiments, cells were treated with 100 or 200 μg/mL PM2.5 for 24 h, with a final DMSO concentration of 0.1% (v/v) in the culture medium. DMSO served as the vehicle control. PM2.5 concentration were selected based on previously published studies using cell models (36). Cell viability was assessed using a CCK-8 assay before experimental analyses. (**Supplementary Figure 1**) All *in vitro* experiments were performed using independent biological replicates, as indicated in the corresponding figure legends.

### 2.4 Masson’s trichrome staining

Mouse tissues were fixed in 4% paraformaldehyde, dehydrated through a graded ethanol series, and embedded in paraffin. Paraffin blocks were sectioned at 4 μm, deparaffinized, rehydrated, and stained using a Masson’s trichrome staining kit (Maixin Biotech, Fuzhou, China) according to the manufacturer’s instructions. Stained sections were examined with a Zeiss Axioscan 7 (Carl Zeiss, Oberkochen, Germany).

### 2.5 Isolation of cytoplasmic and nuclear protein fraction

Cytoplasmic and nuclear extracts were obtained using the NE-PER™ Nuclear and Cytoplasmic Extraction Reagents kit (Thermo Scientific, Waltham, MA, USA) according to the manufacturer’s protocol. Briefly, cells were washed with cold PBS, pelleted at 500 × g for 3 min, and sequentially treated with Cytoplasmic Extraction Reagent I and II. The cytoplasmic fraction was collected after centrifugation at 16,000 × g for 5 min. The remaining pellet was resuspended in Nuclear Extraction Reagent, incubated on ice, and centrifuged to obtain the nuclear fraction.

### 2.6 CCK-8 assay

Cell viability was assessed using a Cell Counting Kit-8 (CCK-8; Dojindo Laboratories, Kumamoto, Japan) according to the manufacturer’s instructions. Briefly, cells were seeded at 7 × 10³/well in 96-well plates, treated with PM2.5 for 24 h, and then incubated with 20 μL of CCK-8 solution for 1–3 h. Absorbance was measured at 450 nm using a microplate reader (Molecular Devices, San Jose, CA, USA), and cell viability was calculated relative to DMSO-treated controls.

### 2.7 Western blotting

Total protein was isolated using radioimmunoprecipitation lysis buffer (RIPA; Thermo Scientific) supplemented with protease and phosphatase inhibitor cocktails (PI; Thermo Scientific). Protein concentrations were determined with a bicinchoninic acid assay kit (BCA; Thermo Scientific). Equal protein amounts (12 μg) were denatured, separated on 10% sulfate-polyacrylamide gel electrophoresis (SDS-PAGE), and transferred to polyvinylidene fluoride membranes (PVDF; Merck, Darmstadt, Germany). Membranes were blocked with 5% non-fat skim milk in Tris-buffered saline solution (10 mM Tris-HCl [pH 7.4] and 0.5 M NaCl) with Tween-20 (0.1% v/v) for 1 h and incubated overnight at 4 °C with the following specific primary antibodies (full list in **Supplementary Table 1**). After washing, membranes were incubated with HRP-conjugated secondary antibodies (anti-mouse or anti-rabbit IgG, 1:3000; Thermo Scientific) for 1 h at 20 °C. Bands were visualized using chemiluminescent substrate (SuperSignal West Pico Plus; Thermo Scientific) and imaged with an ImageQuant LAS 4000 system (General Electric, Chicago, IL, USA). Densitometry was performed using ImageJ software (NIH, Bethesda, Maryland, USA).

### 2.8 Quantitative real time PCR

Total RNA was extracted with TRIzol (Invitrogen), and RNA purity and concentration were assessed using a NanoDrop ND-2000 spectrophotometer (ThermoFisher Scientific). cDNA was synthesized from 1 μg of RNA using the Maxima First Strand cDNA synthesis kit (Thermo Scientific). qPCR was conducted with PowerUp™ SYBR™ Green master mix (Thermo Scientific) on a Step One Plus real-time PCR system (Applied Biosystems, Foster City, USA). Cycling conditions consisted of an initial denaturation at 95 °C for 10 min, followed by 40 cycles of denaturation at 95 °C for 15 s, and annealing and extension at 60 °C for 1 min. Gene expression was normalized to GAPDH and analyzed using the 2-ΔΔCT method (37). The sequences of the PCR primers are provided in **Supplementary Table 1**.

### 2.9 Statistical analysis

Data are presented as mean ± SD. Statistical tests were performed using GraphPad Prism 5 (GraphPad Software, San Diego, CA, USA). The Shapiro–Wilk test assessed normality, and Levene’s test evaluated variance homogeneity. Comparisons between two groups were made using Student’s t-test or the Mann–Whitney U test, as appropriate. For more than two groups, one-way ANOVA followed by Tukey’s *post hoc* test was used. For the CCK-8 assay shown in **Supplementary Figure 1**, the dataset was reanalyzed using the Kruskal–Wallis test followed by Dunn’s multiple-comparison test. Differences were considered statistically significant at p < 0.05.

## 3 Results

### 3.1 PM2.5 exposure induces postpartum hair loss in mouse scalp

Postpartum hair loss was markedly aggravated in mice exposed to PM2.5. Compared with controls, the PM2.5-treated animals exhibited pronounced scalp hair loss.

**Figure 2:**
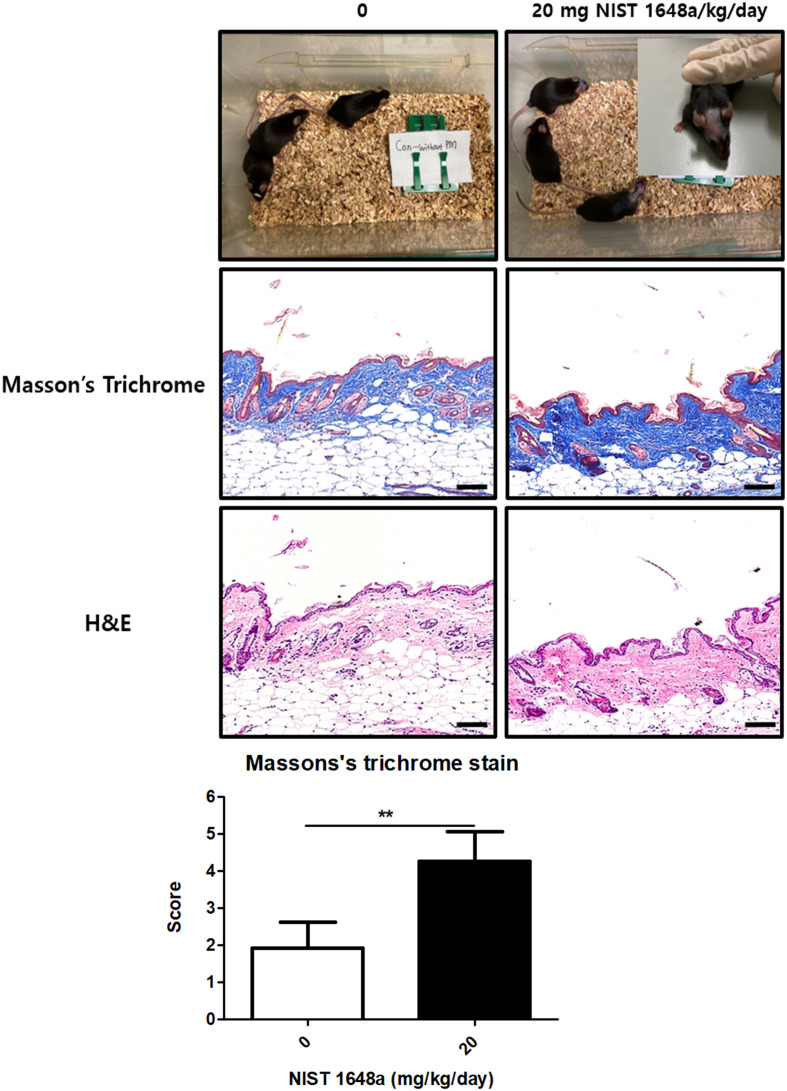
PM2.5 exposure induces hair loss in postpartum mice. Representative images of postpartum mice are shown. Scalp tissues were subjected to H&E and Masson’s trichrome staining to assess histological alterations. Three mice are shown per group in the cage images. In the PM2.5-exposed group, hair loss was observed in two of the three mice shown. One representative mouse with evident hair loss is shown at higher magnification. Quantitative analyses were performed using scalp tissues from eight mice per group. (Scale bar = 200 μm) The data represent the mean ± SD. (N = 8, **p < 0.01).

Histopathological evaluation revealed several structural alterations in the PM2.5-treated scalp, including a thicker stratum corneum, downward displacement of hair follicles into the deeper dermis, and a reduction in follicle density. Masson’s trichrome staining further demonstrated an accumulation of collagen fibers within the dermal layer (1.92 ± 0.70 vs. 4.26 ± 0.80, *p* = 0.0094). While hair follicles in control mice were located adjacent to the epidermis, those in PM2.5-exposed mice were fewer in number and positioned deeper within the dermis, increasing their distance from the epidermal surface. Dermal thickness also showed an upward trend in the PM2.5 group (**Figure 2**). Collectively, these morphological changes suggest that PM2.5 exposure contributes to postpartum hair loss by inducing dermal thickening, promoting follicular regression, and enhancing collagen deposition. Notably, visible hair loss occurred exclusively in postpartum PM2.5-exposed animals, whereas no hair loss was observed in any non-pregnant group regardless of PM2.5 treatment (**Supplementary Figure 2**). Hair loss was predominantly observed in the scalp region and was not distributed across the entire body. Hair loss was assessed through both gross photographic observation and histological analysis. Based on these findings, subsequent analyses focused on postpartum mice, with comparisons made between postpartum control and PM2.5- exposed groups.

### 3.2 PM2.5 exposure enhances inflammatory responses in mouse scalp tissue

**Figure 3:**
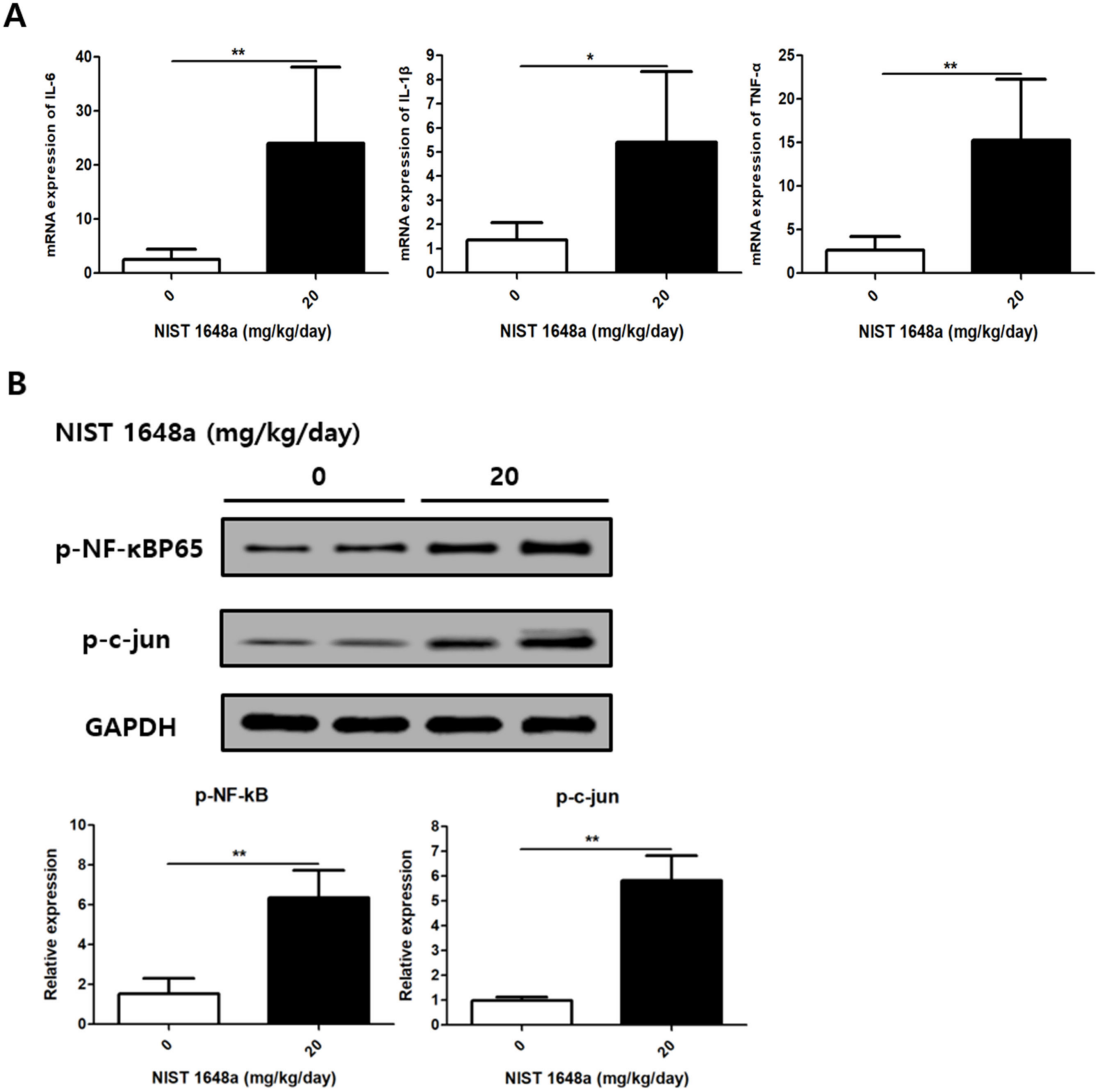
PM2.5 exposure increases inflammatory cytokines in mouse scalp. **(A)** Relative mRNA expression levels of cytokines (IL-6, IL-1β, and TNF-α) were analyzed. **(B)** Relative protein levels of inflammatory markers (p-NF-κB and p-c-Jun) were analyzed. The data represent the mean ± SD. (N = 8, *p < 0.05, **p < 0.01).

qPCR analysis revealed that PM2.5-treated mice expressed significantly higher mRNA levels of IL-6, IL-1β, and TNF-α than controls (IL-6: 2.52 ± 1.89 vs. 23.98 ± 14.16, *p* = 0.0042; IL-1β: 1.36 ± 0.7 vs. 5.41 ± 2.92, *p* = 0.0186, TNF-α: 2.63 ± 1.57 vs. 15.29 ± 6.97, *p* = 0.0036) (**Figure 3A**). These findings indicate that PM2.5 exposure induces a pronounced inflammatory response in postpartum mouse scalp tissue.

Western blot analysis further demonstrated increased levels of p-NF-κB p65 and p-c-Jun in the PM2.5-exposed group (p-NF-kB: 1.52 ± 0.77 vs. 6.36 ± 1.38, p = 0.0038; p-c-jun: 0.98 ± 0.14 vs. 5.82 ± 1, p = 0.0039) (**Figure 3B**). This activation of NF-κB and AP-1 signaling pathways supports the notion that PM2.5 exposure promotes pro-inflammatory signaling in tissue.

### 3.3 PM2.5 exposure promotes apoptotic and oxidative stress signaling in mouse scalp

**Figure 4:**
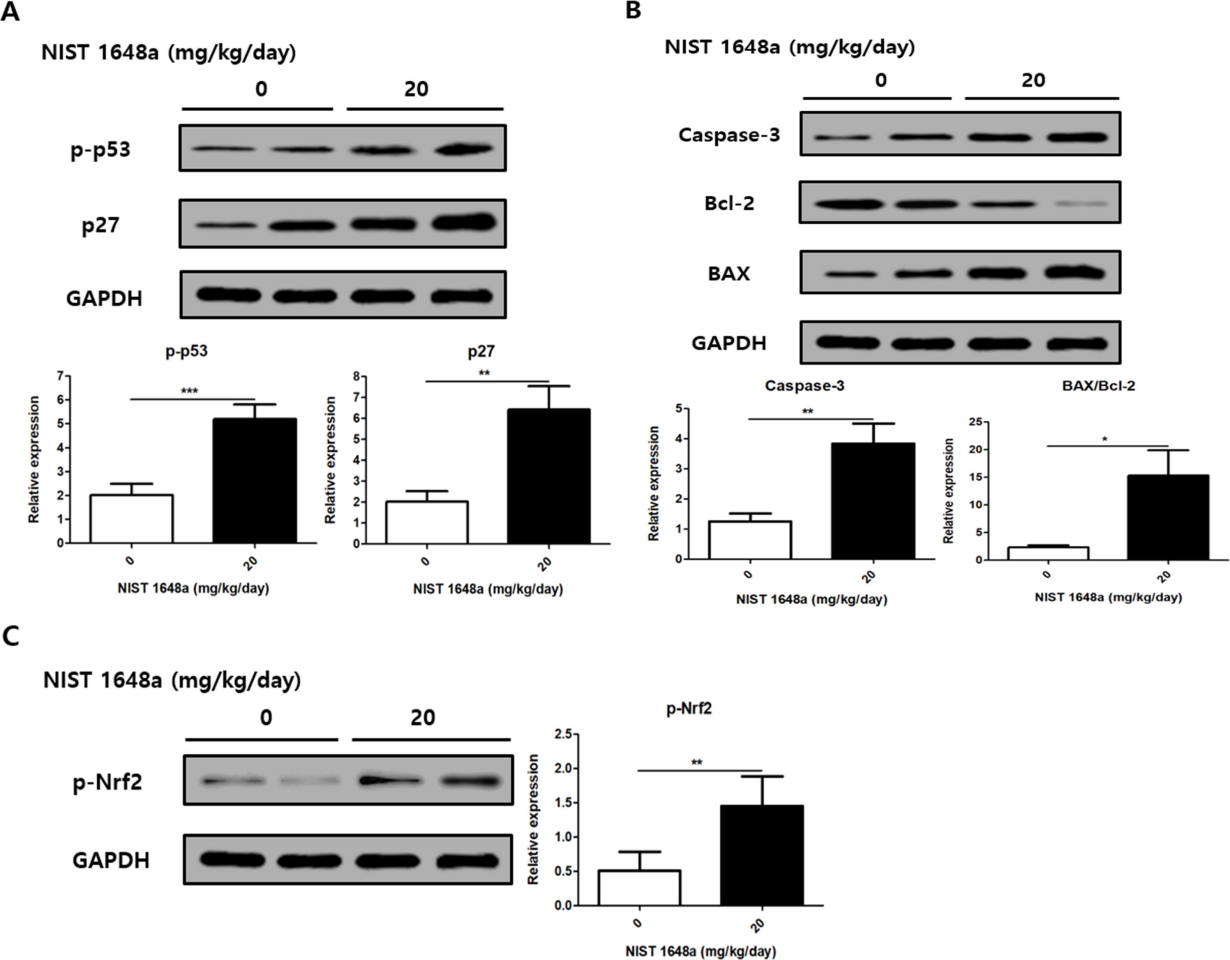
PM2.5 exposure induces apoptotic and induces oxidative stress pathways in mouse scalp. **(A)** Relative protein levels of p-p53 and p27 were analyzed. **(B)** Relative protein levels of Caspase-3 and the BAX/Bcl-2 ratio were analyzed. **(C)** The relative protein levels of p-Nrf2 were analyzed as a marker of oxidative stress. The data represent the mean ± SD. (N = 8, *p < 0.05, **p < 0.01, ***p<0.001).

The protein levels of p-p53 and p27 were significantly elevated in PM2.5-exposed scalp tissue compared to controls (p-p53: 2 ± 0.48 vs. 5.2 ± 0.6, p = 0.0005; p27: 2.03 ± 0.48 vs. 6.42 ± 1.12, p = 0.0033) (**Figure 4A**). These changes suggest activation of p53–related signaling in response to PM2.5 exposure. The BAX/Bcl-2 ratio and Caspase-3 levels were also increased in the PM2.5 group (Caspase-3: 1.25 ± 0.27 vs. 3.83 ± 0.67, p = 0.0046; BAX/Bcl-2: 2.3 ± 0.41 vs. 15.35 ± 4.56, p = 0.0222), indicating enhanced apoptotic signaling in the scalp tissue (**Figure 4B**). In addition, oxidative stress was assessed by measuring p-Nrf2 levels in mouse scalp tissues. PM2.5 exposure significantly increased p-Nrf2 expression compared with controls (0.50 ± 0.28 vs. 1.45 ± 0.43, p = 0.0018), indicating activation of the oxidative stress response (**Figure 4C**).

### 3.4 PM2.5 exposure increases fibrosis-associated markers

**Figure 5:**
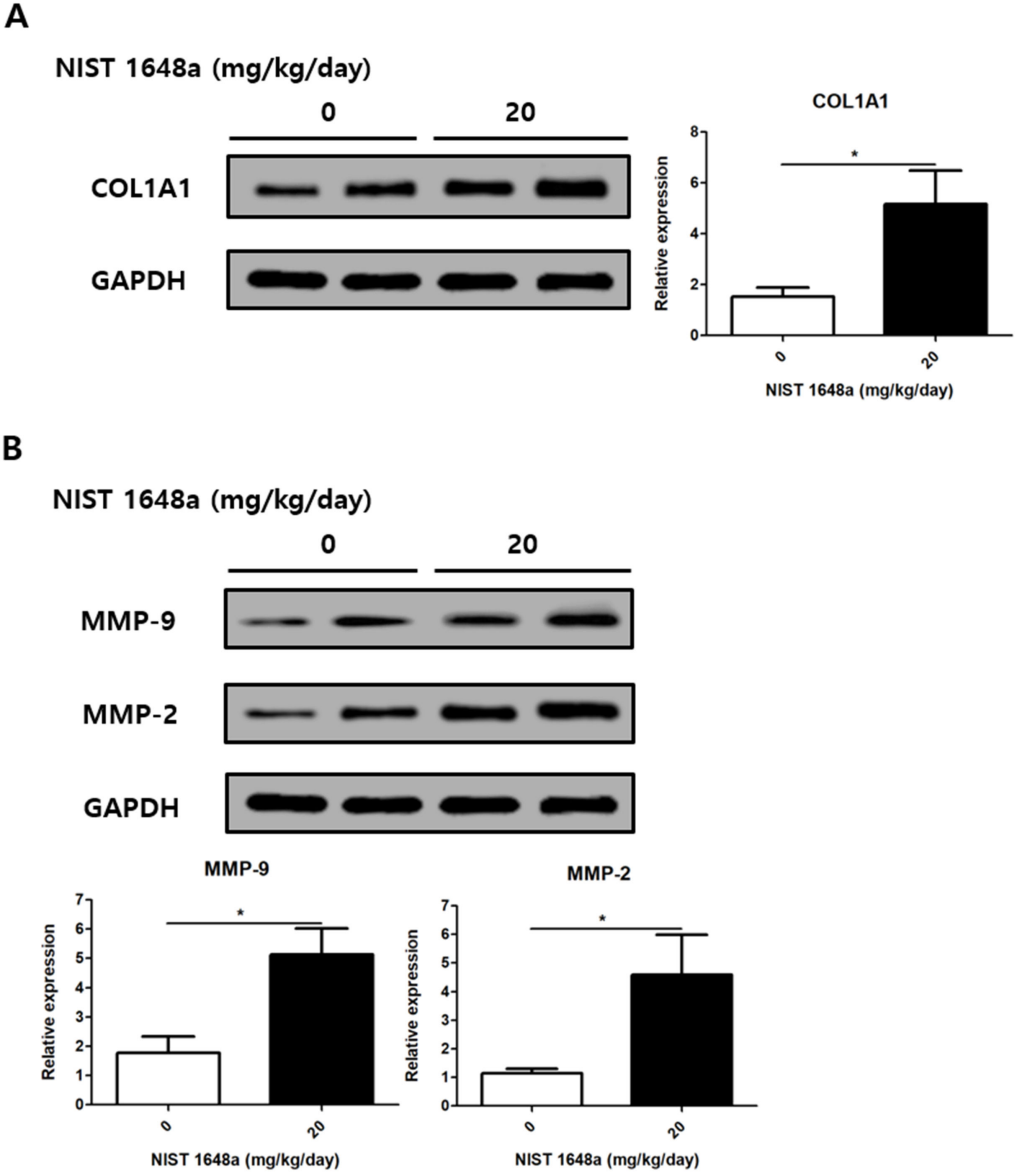
PM2.5 exposure accumulates fibrosis in mouse scalp. **(A)** Relative protein levels of COL1A1 were analyzed. **(B)** Relative protein levels of MMP-9 and MMP-2 were analyzed. The data represent the mean ± SD. (N = 8, *p < 0.05).

COL1A1 protein expression was markedly higher in PM2.5-treated mice (1.52 ± 0.37 vs. 5.17 ± 1.31, p = 0.0237) (**Figure 5A**). This increase indicates enhanced collagen deposition in the scalp tissue following PM2.5 exposure. The expression levels of MMP-2 and MMP-9 levels were also increased in the PM2.5 group (MMP-2: 1.14 ± 0.16 vs. 4.6 ± 1.4, p = 0.0359; MMP-9: 1.77 ± 0.57 vs. 5.12 ± 0.9, p = 0.0166) (**Figure 5B**). These findings suggest that PM2.5 exposure induces extracellular matrix remodeling and promotes fibrotic changes in the scalp.

### 3.5 PM2.5 exposure downregulates hormone receptors and HFSCs markers

**Figure 6:**
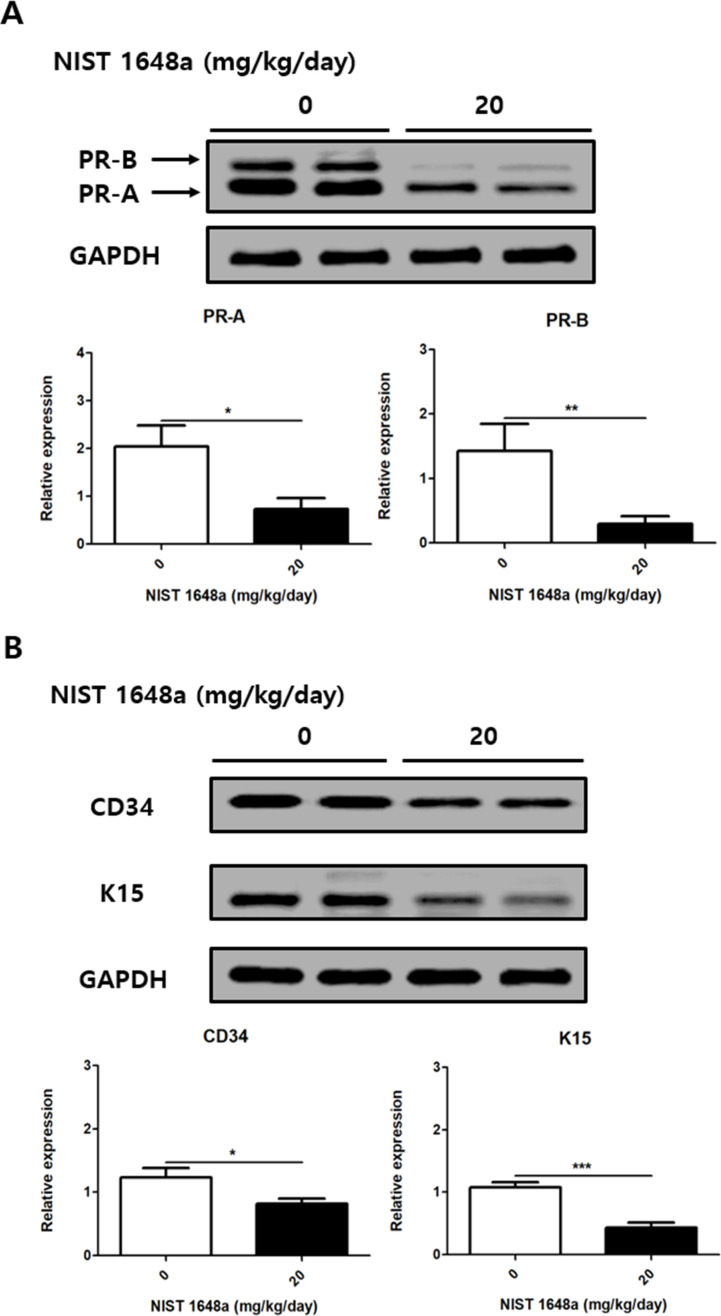
PM2.5 exposure decreases progesterone receptor and HFSCs markers in mouse scalp. **(A)** Relative protein levels of PR-A and PR-B were analyzed. **(B)** Relative protein levels of CD34 and K15 were analyzed. The data represent the mean ± SD. (N = 8, *p < 0.05, **p < 0.01, ***p < 0.001).

PM2.5 exposure reduced progesterone receptor isoforms PR-A and PR-B in mouse scalp tissues. (PR-A: 2.04 ± 0.45 vs. 0.73 ± 0.22, p = 0.0703; PR-B: 1.42 ± 0.42 vs. 0.3 ± 0.12, p = 0.0064) (**Figure 6A**). This reduction suggests altered hormone-related signaling in the scalp following PM2.5 exposure. HFSCs markers CD34 and K15 were also significantly reduced in mouse scalp tissues (CD34: 1.24 ± 0.14 vs. 0.82 ± 0.09, p = 0.0286; K15: 1.08 ± 0.08 vs. 0.43 ± 0.08, p = 0.0004) (**Figure 6B**). These findings indicate a reduction in markers associated with hair follicle stem cells in response to PM2.5 exposure.

### 3.6 PM2.5 exposure induces inflammatory cytokine in human HaCaT cells and fibroblasts

Cell viability was first assessed using the CCK-8 assay, which showed a concentration-dependent decrease in both HaCaT cells and fibroblasts following PM2.5 exposure (**Supplementary Figure 1**).

**Figure 7:**
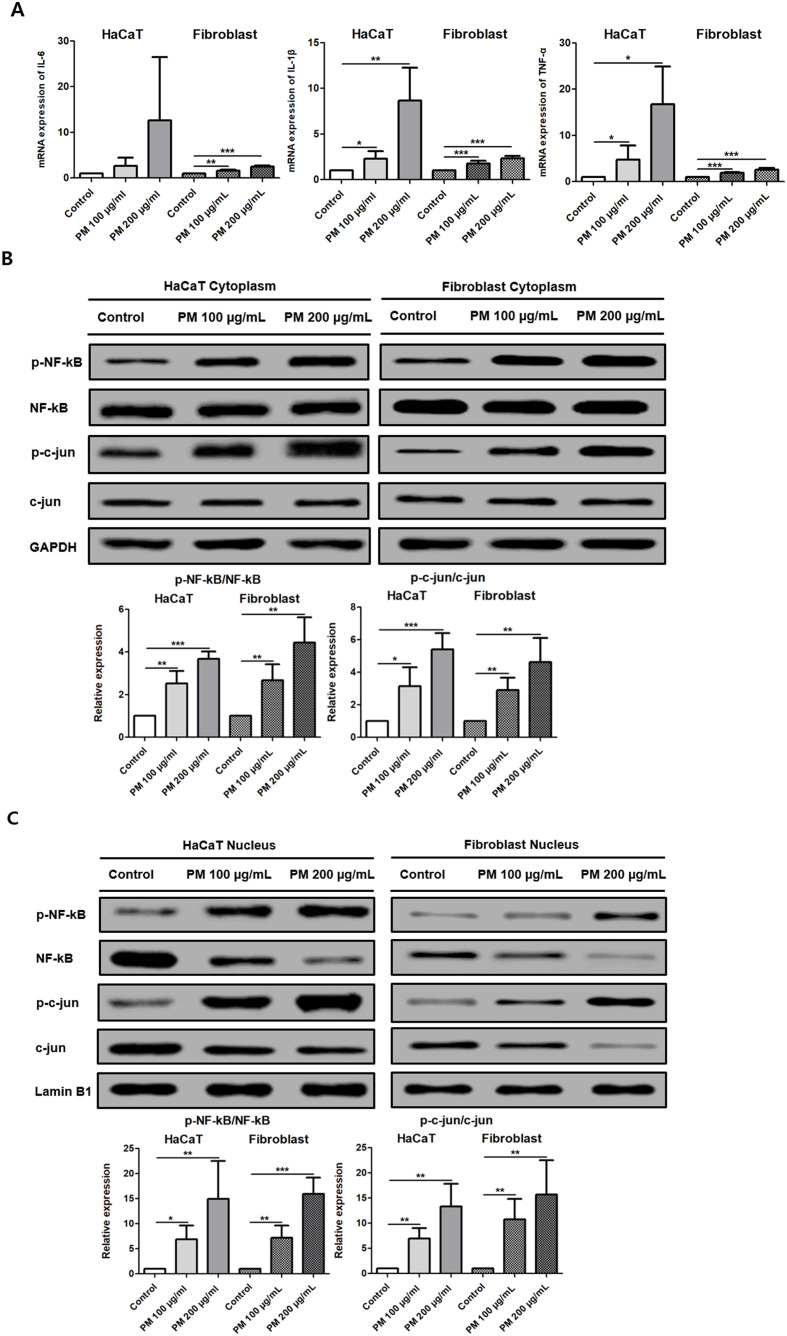
PM2.5 exposure increases inflammatory cytokine expression in HaCaT cells and fibroblasts. **(A)** Relative mRNA expression levels of cytokines (IL-6, IL-1β, and TNF-α) were analyzed. **(B)** Relative protein levels of inflammatory markers (p-NF-κB and p-c-jun) were analyzed in the cytoplasmic fraction. **(C)** Relative protein levels of inflammatory markers (p-NF-κB and p-c-jun) were analyzed in the nuclear fraction. Eight independent biological replicates were analyzed for qPCR, with each sample measured in triplicate. Western blot analysis was performed using eight independent biological replicates. The data represent the mean ± SD. (*p < 0.05, **p < 0.01, ***p<0.001).

PM2.5 exposure for 24 h increased inflammatory cytokine expression in both HaCaT cells and fibroblasts. In HaCaT cells, IL-1β mRNA levels were significantly elevated after PM2.5 treatment (1 vs. 2.31 ± 0.8, p = 0.0211 at 100 μg/mL; 8.65 ± 3.62, p = 0.0091 at 200 μg/mL). TNF-α mRNA levels were also increased (1 vs. 4.77 ± 1.36, p = 0.0498 at 100 μg/mL; 16.77 ± 8.13, p = 0.0123 at 200 μg/mL). In fibroblasts, PM2.5 exposure significantly increased the mRNA expression of IL-6 (1 vs. 1.59 ± 0.23, p = 0.0043 at 100 μg/mL; 2.48 ± 0.11, p = 0.0002 at 200 μg/mL), IL-1β (1 vs. 1.76 ± 0.13, p = 0.0041 at 100 μg/mL; 2.35 ± 0.1, p = 0.0002 at 200 μg/mL), and TNF-α (1 vs. 1.88 ± 0.17, p = 0.0003 at 100 μg/mL; 2.58 ± 0.33, p = 0.0004 at 200 μg/mL) (**Figure 7A**). These findings indicate that PM2.5 exposure induces a robust inflammatory response in both cell types.

Western blot analysis further demonstrated increased ratios of phosphorylated to total NF-κB and c-jun in both cytoplasmic and nuclear fractions following PM2.5 exposure. In HaCaT cells, p-NF-κB/NF-κB levels were increased in the cytoplasm (1 vs. 2.53 ± 0.57, p = 0.004 at 100 μg/mL; 3.68 ± 0.33, p = 0.0001 at 200 μg/mL) and nucleus (1 vs. 6.88 ± 2.72, p = 0.0085 at 100 μg/mL; 14.97 ± 7.55, p = 0.0144 at 200 μg/mL). Similar increases were observed for p-c-jun/c-jun in the cytoplasm (1 vs. 3.14 ± 1.17, p = 0.0148 at 100 μg/mL; 5.4 ± 1, p = 0.0006 at 200 μg/mL) and nucleus (1 vs. 6.95 ± 2.06, p = 0.003 at 100 μg/mL; 13.3 ± 4.51, p = 0.0037 at 200 μg/mL) (**Figures 7B, C**). In fibroblasts, cytoplasmic p-NF-κB/NF-κB levels were increased (1 vs. 2.67 ± 0.76, *p* = 0.0082 at 100 μg/mL, 4.45 ± 1.17, *p* = 0.0028 at 200 μg/mL), and nuclear levels were similarly elevated (1 vs. 7.21 ± 2.4, *p* = 0.0044 at 2.5 100 μg/mL, 15.94 ± 3.26, *p* = 0.0005 at 200 μg/mL). The p-c-jun/c-jun ratios were also increased in both the cytoplasm (1 vs. 2.9 ± 0.75, *p* = 0.0048 at 100 μg/mL, 4.16 ± 1.95, *p* = 0.0055 at 200 μg/mL), and (1 vs. 10.75 ± 4.08, *p* = 0.0059 at 100 μg/mL, 15.69 ± 6.8, *p* = 0.0085 at 200 μg/mL) nucleus (**Figures 6B, C**). Together, these molecular changes suggest activation of NF-κB and c-Jun–related inflammatory signaling pathways in both HaCaT cells and fibroblasts following PM2.5 exposure, consistent with previous reports (38, 39).

### 3.7 PM2.5 exposure triggers apoptosis and suppresses proliferation in human HaCaT cells and fibroblasts

PM2.5 exposure for 24 h altered apoptotic and proliferative signaling pathways in both HaCaT cells and fibroblasts.

In HaCaT cells, PM2.5 exposure significantly increased the p-p53/p53 ratio (1 vs. 1.88 ± 0.37, p = 0.006 at 100 μg/mL, 3.47 ± 0.38, p = 0.0001 at 200 μg/mL), p27 expression (1 vs. 2.17 ± 0.26, p = 0.0006 at 100 μg/mL, 4.86 ± 0.63, p = 0.0001 at 200 μg/mL), Caspase-3 levels (1 vs. 3.02 ± 0.0006, p = 0.02 at 100 μg/mL, 3.69 ± 0.32, p = 0.0001 at 200 μg/mL), and the BAX/Bcl-2 ratio (1 vs. 4.09 ± 1.76, p = 0.0173 at 100 μg/mL, 14.89 ± 2.28, p = 0.0002 at 200 μg/mL).

**Figure 8:**
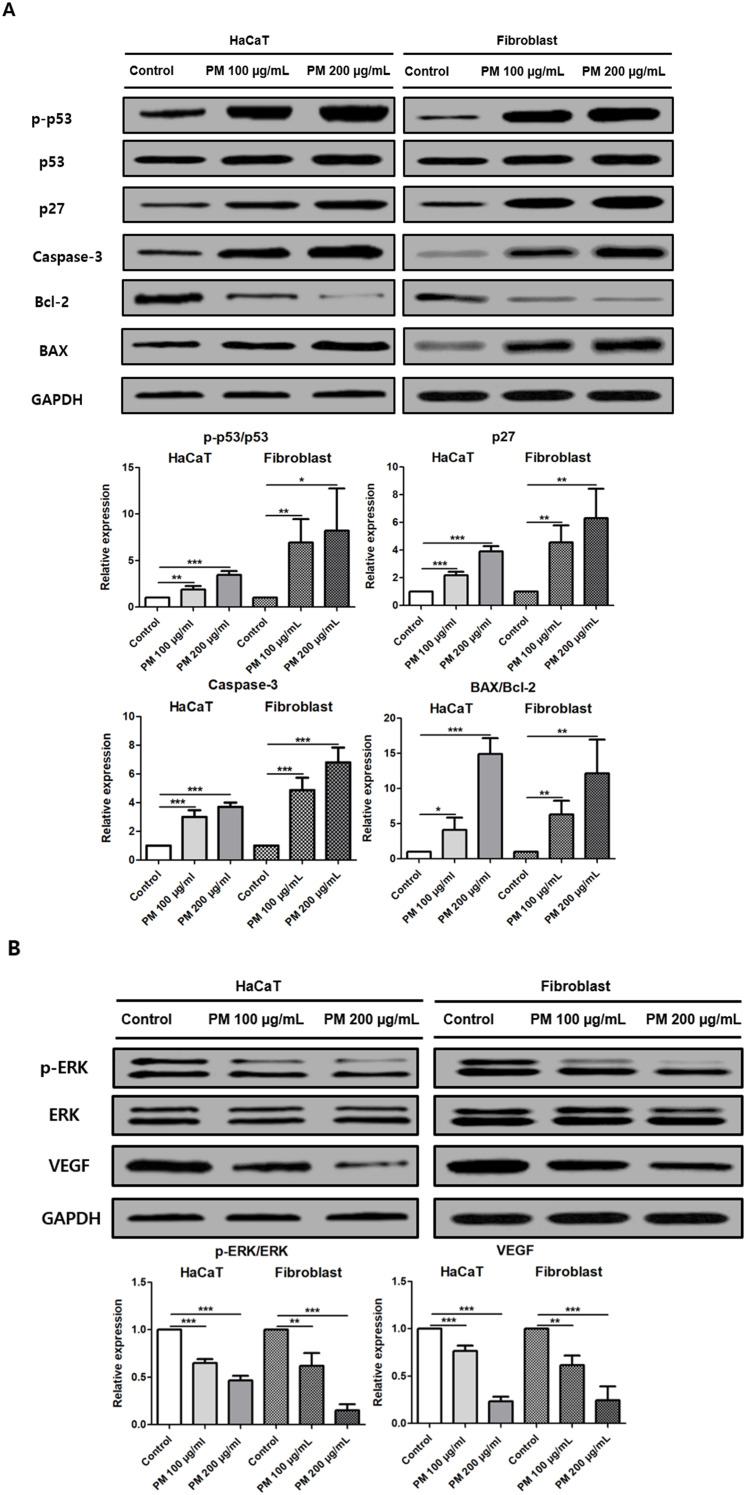
PM2.5 exposure alters apoptotic and proliferative signaling pathway in HaCaT cells and fibroblasts. **(A)** Relative protein levels of apoptosis-related markers (p-p53, p53, p27, Caspase-3, Bcl-2, and BAX) were analyzed. **(B)** Relative protein levels of proliferation-related markers (p-ERK, ERK, and VEGF). Western blot analyses were performed using eight independent biological replicates. The data represent the mean ± SD. (*p < 0.05, **p < 0.01, ***p<0.001).

Fibroblasts exhibited similar increases in the p-p53/p53 (1 vs. 6.94 ± 2.53, p = 0.0063 at 100 μg/mL, 8.21 ± 4.56, p = 0.024 at 200 μg/mL), p27 (1 vs. 4.55 ± 1.22, p = 0.0029 at 100 μg/mL, 6.31 ± 2.11, p = 0.0049 at 200 μg/mL), Caspase-3 (1 vs. 4.87 ± 0.87, p = 0.0006 at 100 μg/mL, 6.81 ± 1.01, p = 0.0002 at 200 μg/mL), and the BAX/Bcl-2 ratio (1 vs. 6.29 ± 1.98, p = 0.004 at 100 μg/mL, 12.18 ± 4.76, p = 0.0063 at 200 μg/mL) (**Figure 8A**).

Conversely, proliferative signaling markers were significantly reduced by PM2.5 exposure. In HaCaT cells, the p-ERK/ERK ratio and VEGF expression were decreased following PM2.5 treatment (p-ERK/ERK: 1 vs. 0.65 ± 0.04, p = 0.0001 at 100 μg/mL, 0.47 ± 0.05, p = 0.0001 at 200 μg/mL, VEGF: 1 vs. 0.77 ± 0.06, p = 0.0008 at 100 μg/mL, 0.23 ± 0.05, p = 0.0001 at 200 μg/mL).

Similarly, fibroblasts exposed to PM2.5 showed a significant decrease in the p-ERK/ERK ratio and VEGF expression (p-ERK/ERK: 1 vs. 0.62 ± 0.13, p = 0.0032 at 100 μg/mL, 0.15 ± 0.06, p = 0.0001 at 200 μg/mL, VEGF: 1 vs. 0.62 ± 0.1, p = 0.001 at 100 μg/mL, 0.25 ± 0.14, p = 0.0003 at 200 μg/mL) (**Figure 8B**).

Together, these results indicate that PM2.5 exposure promotes apoptotic signaling while suppressing proliferation-related pathways in both HaCaT cells and fibroblasts, consistent with previous reports (27, 40).

## 4 Discussion

This study investigated the effects of PM2.5 exposure on postpartum hair loss and explored potential underlying mechanisms using both *in vivo* and *in vitro* models. PM2.5 exposure exacerbated hair loss in postpartum mice and was associated with alterations in inflammatory responses, apoptosis, fibrosis, and hormone-related signaling pathways. Notably, these changes were not observed in non-pregnant mice, suggesting a specific vulnerability during the postpartum period. In particular, our findings indicate that PM2.5 exposure is associated with changes in progesterone receptor expression and hair follicle stem cell-related markers, which have not been previously reported in the context of postpartum hair loss. While previous studies have described the effects of PM2.5 on skin inflammation, the mechanistic link between PM2.5 exposure and postpartum hair loss remains poorly understood.

To model physiologically relevant systemic exposure to PM2.5, intranasal administration was selected for the *in vivo* experiments because inhalation is the primary route of PM2.5 entry into the body and can induce systemic inflammatory and hormonal alterations. Although the skin is an important interface with the external environment, this study focused on systemic effects rather than direct topical exposure.

HaCaT cells and fibroblasts were used as alternative models due to the limitation of not being able to DP cells in human scalp skin. Based on previous studies (41, 42), the use of HaCaT cells and fibroblasts cells as alternative models is an appropriate approach to study signaling between skin cells and changes in factors associated with hair loss.

The PM2.5 dose used in this study was selected based on previously published *in vivo* studies demonstrating PM2.5-induced inflammatory responses in animal models, while the *in vitro* concentrations were based on studies using skin cell models reporting oxidative stress and inflammatory effects (30, 31, 36). These concentrations were chosen to ensure detectable molecular and cellular changes under the present experimental conditions. Although lower concentrations of PM2.5 may also be relevant to human exposure, the present study focused on identifying mechanistic alterations under conditions that reliably induce measurable responses. Evaluation of lower, more physiologically relevant doses will be an important topic for future studies.

Increased inflammatory cytokines (IL-6, IL-1β, and TNF-α) in mouse scalp indicate that PM2.5 triggers inflammatory conditions capable of activating p-NF-kB and p-c-jun pathways, consistent with previous dermatological models (43, 44).

Apoptotic signaling was also increased such as p-p53, p27, Caspase-3, and BAX/Bcl-2 ratio. PM2.5 triggers cellular stress within hair follicles, leading to increased apoptosis and follicular damage, disrupting the normal hair growth cycle and accelerating hair loss (13, 45, 46).

Fibrosis represents another pathological change associated with PM2.5, as evidenced by increased collagen deposition and elevated expression of metalloproteinases (MMP-2, -9) observed in this study. Fibrotic remodeling has been implicated in various dermatological conditions, including scarring alopecia (47, 48). Previous studies have shown that excessive extracellular matrix deposition and dysregulated matrix remodeling can disrupt the structural integrity of hair follicles and surrounding scalp tissue, ultimately contributing to hair loss (49, 50).

The altered hormonal signaling observed in this study included reduced expression of progesterone receptor isoforms (PR-A and PR-B) in the scalp of PM2.5-exposed postpartum mice. Progesterone is known to play an important role in regulating hair growth and hair cycle progression, and the physiological decline in progesterone levels after childbirth is considered a key mechanism underlying postpartum hair loss (51–53). Therefore, disruption of progesterone signaling by PM2.5 exposure may further exacerbate hair loss by promoting the premature transition of hair follicles from the anagen phase (24).

HFSCs play a crucial role in hair generation, and well-established HFSCs markers include CD34, K15, Lgr5, Sox9 and Lhx2 (54, 55). Among these, CD34 and K15 serve as specific markers for hair follicle stem cells in mice (29). CD34 plays an important role in maintaining HFSCs function and contributes to hair follicle generation (56). K15 is a key structural and functional protein expressed in the bulge region of hair follicles, where it contributes to stem cell integrity and niche stability (57, 58). Loss of K15 has been shown to impair HFSCs maintenance and disrupt normal hair follicle cycling, resulting in premature hair thinning, as demonstrated in K15 knockout mice (59–61). The expression of CD34 and K15 was reduced in the scalp of PM2.5-exposed postpartum mice, suggesting that PM2.5 exposure may impair HFSCs maintenance and function, thereby accelerating postpartum hair loss. Although we did not directly assess HFSCs self-renewal or differentiation capacity, the reduced expression of these markers raises the possibility that PM2.5 exposure disrupts stem cell homeostasis and regenerative potential.

Hair follicles undergo cyclic phases consisting of anagen, catagen, and teloegen (62). Environmental exposures, including PM2.5, have the potential to disrupt the normal hair follicle cycle (36). PM2.5 exposure induced histologic changes in the mouse scalp that included deeper migration of hair follicles into the dermis and a decrease in follicle number. Collagen accumulation observed through Masson’s trichrome staining indicates that PM2.5 induces scalp fibrosis, which may interfere with hair follicle regeneration (63, 64). The hair shedding observed following PM2.5 exposure resembles postpartum hair loss associated with a rapid decrease in progesterone levels, suggesting that PM2.5 disrupts hormonal signaling pathways and disturb the hair cycle (24).

The observed hair loss in the scalp suggests that PM2.5 exposure may induce hormonal imbalances, leading to a premature transition of hair follicles from anagen to telogen phase, which promotes hair loss (36, 65). Histological analysis of our samples further supported these findings. Specifically, PM-exposed scalps in this study showed a thicker stratum corneum, downward migration of hair follicles into the dermis, reduced follicle density, and increased dermal collagen content.

Exposure to PM2.5 resulted in a reduction of p-ERK and VEGF expression in both HaCaT cells and fibroblasts. The ERK signaling pathway has been shown to enhance the proliferation and migration of DP cells, which are important role in hair follicle regeneration and growth (66). ERK activation has been reported to be an important pathway associated with DP cell proliferation and hair growth (67), and it also plays an important role in regulating cellular proliferation and differentiation processes (68). VEGF promotes hair growth by enhancing microvascular permeability and stimulating angiogenesis (69). Previous studies have shown reduced VEGF expression in baldness, and our findings similarly demonstrated a decline in VEGF expression (70).

The results of this study indicate that PM2.5 exposure contributes to postpartum hair loss through inflammation, apoptosis, fibrosis, altered hormone signaling, and a reduction in HFSC-related markers. While similar molecular mechanisms have been reported in other tissues (30), this study highlights the specific impact of PM2.5 on the postpartum scalp. Although inflammatory changes were consistently demonstrated by qPCR and western blot analyses, cytokine concentrations were not directly quantified using ELISA, and functional rescue experiments using antioxidants or anti-inflammatory agents were not performed. These approaches would provide stronger mechanistic validation and should be addressed in future studies. In addition, it should be noted that the PM2.5 used in this study was a standardized reference material (NIST^®^ SRM^®^ 1648a) with a certificate of analysis specifying its composition, and the potential contribution of trace endotoxins or heavy-metal contaminants to the observed inflammatory responses cannot be completely excluded. Therefore, caution is warranted when applying these findings to real-world environmental PM2.5 exposure. Direct quantitative comparison between *in vivo* doses and *in vitro* concentrations is challenging due to differences in exposure routes, metabolism, systemic distribution, and the experimental conditions in this study were designed to investigate mechanistic responses rather than to replicate exact human exposure levels. Although mice may exhibit grooming-related fur removal behaviors, no evidence of aggressive grooming or dominance-related hair loss was observed during daily monitoring. While the doses used are higher than typical environmental exposure, they are commonly applied in experimental models to elicit measurable biological effects. Since PM2.5 exposure was initiated prior to conception, it is possible that pre-existing hormonal alterations may have contributed to the observed phenotype. However, hair loss was observed exclusively in postpartum mice exposed to PM2.5 and not in non-pregnant mice, suggesting that the postpartum physiological state plays a key role. Further studies evaluating hormonal profiles will be necessary to clarify this relationship. Overall, these findings provide insight into the potential role of PM2.5 as a contributing factor to postpartum hair loss while highlighting important areas for further investigation.

## 5 Conclusion

The results suggest that PM2.5 exposure contributes to postpartum hair loss by increasing inflammatory cytokine levels, promoting apoptosis and fibrosis, and reducing progesterone receptor expression and hair follicle stem cell–related markers. We observed detrimental effects of PM2.5 on postpartum scalp health, potentially through disruption of the hair growth cycle and impairment of follicular regenerative capacity. Collectively, these findings suggest a previously unrecognized mechanism by which environmental PM2.5 exposure may contribute to postpartum hair loss. Future studies using additional experimental systems, including human-derived models, will be helpful to further validate and extend these findings. Moreover, investigation of hair cycle–related markers may provide deeper insight into the effects of PM2.5 on hair follicle biology.

## Data Availability

The raw data supporting the conclusions of this article will be made available by the authors, without undue reservation.
