# Supplementary material for: Particulate matter exposure induces maternal scalp hair loss after birth in C57/B6 mouse via alteration of inflammatory and apoptotic pathways
Source: Front Endocrinol (Lausanne). 2026 May 8;17:1766198. doi: 10.3389/fendo.2026.1766198 (PMC13193834; doi:10.3389/fendo.2026.1766198)
Supplement: Supplementary file 1 [file Table1.docx]

**Particulate matter exposure induces maternal scalp hair loss after birth in C57/B6 mouse via alteration of inflammatory and apoptotic pathways**

Gee Soo Jung, Min Jung Lee, Wooseok Im, Hyemin Park, Inha Lee, Jae Hoon Lee, HYENO HO KU, Sang Eun Lee, SiHyun Cho, and Young Sik Choi

**Supplementary Table S1** : Primary antibodies for western blotting

**Supplementary Table S2** : Quantitative RT–PCR human and mouse primer sequences

**Supplementary Figure S1** : Cell proliferation was measured using the CCK-8 assay.

**Supplementary Figure S2** : Representative images of control or PM2.5 exposure in non-birth and birth mice.

**Supplementary Figure S3** : Original western blot images corresponding to the main figures.

**Supplementary Table S1. Primary antibodies for western blotting**

| **Primary antibody** | **Dilution** | **manufacturer** |
| --- | --- | --- |
| Phospho-p44/42 mitogen-activated protein kinases (MAPK) (p-ERK 1/2) | 1:500 | Cell Signaling Technology, Danvers, MA, USA |
| p44/42 MAPK (ERK 1/2) | 1:500 | Cell Signaling Technology, Danvers, MA, USA |
| Phospho-tumor protein p53 (p-p53) | 1:1000 | Santa Cruz, Dallas, TX, USA |
| tumor protein p53 (p53) | 1:1000 | Santa Cruz, Dallas, TX, USA |
| Phospho-nuclear factor (NF)-κB p65 (p- NF-κB) | 1:1000 | Cell Signaling Technology, Danvers, MA, USA |
| nuclear factor (NF)-κB p65 (NF-κB) | 1:1000 | Cell Signaling Technology, Danvers, MA, USA |
| Phospho-transcription factor AP-1 subunit c-Jun (p-c-jun) | 1:1000 | Cell Signaling Technology, Danvers, MA, USA |
| Transcription factor AP-1 subunit c-Jun (c-jun) | 1:1000 | Cell Signaling Technology, Danvers, MA, USA |
| Phosphorylated nuclear factor erythroid 2 related factor 2 (p-Nrf2) | 1:1000 | Invitrogen, Thermo Fisher Scientific, Waltham, MA, USA |
| Caspase 3 | 1:200 | Santa Cruz, Dallas, TX, USA |
| B-cell lymphoma 2 (Bcl-2) | 1:500 | Santa Cruz, Dallas, TX, USA |
| Bcl-2 Associated X-protein (BAX) | 1:500 | Santa Cruz, Dallas, TX, USA |
| progesterone receptor (PR) | 1:200 | Santa Cruz, Dallas, TX, USA |
| Matrix metalloproteinase-9 (MMP-9) | 1:1000 | Santa Cruz, Dallas, TX, USA |
| Matrix metalloproteinase-2 (MMP-2) | 1:1000 | Santa Cruz, Dallas, TX, USA |
| Cyclin-dependent kinase inhibitor 1B (p27) | 1:500 | Cell Signaling Technology, Danvers, MA, USA |
| collagen type I alpha 1 chain (COL1A1) | 1:1000 | Abcam, Cambridge, UK |
| Lamin B1 | 1:1000 | Santa Cruz, Dallas, TX, USA |
| Vascular endothelial growth factor (VEGF) | 1:200 | Santa Cruz, Dallas, TX, USA |
| Cluster of differentiation 34 (CD34) | 1:200 | Santa Cruz, Dallas, TX, USA |
| Cytokeratin 15 (K15) | 1:200 | Santa Cruz, Dallas, TX, USA |
| glyceraldehyde-3-phosphate dehydrogenase (GAPDH) | 1:2000 | Santa Cruz, Dallas, TX, USA |

**Supplementary Table S2. Quantitative RT–PCR human and mouse primer sequences**

| **Gene** | **Direction** | **Primer sequence** (5′ → 3′) |
| --- | --- | --- |
| *Human IL-6* | F | GAACTCCTTCTCCACACAAGCG  TTTTCTGCCAGTGCCTCTTT |
|  | R |  |
| *Human IL-1β* | F | AAGTGTCTGAAGCAGCCATGGCA  TGAAGCCCTTGCTGTAGTGGTGGT |
|  | R |  |
| *Human TNF-α* | F | TGCCTGCTGCACTTTGGAGTGAT  TGGTTATCTCTCAGCTCCACGCCAT |
|  | R |  |
| *Human GAPDH* | F | CCCTTCATTGACCTCAACTACATG  TGGGATTTCCATTGATGACAAGC |
|  | R |  |
| *Mouse IL-6* | F | TACCACTTCACAAGTCGGAGGC  CTGCAAGTGCATCATCGTTGTTC |
|  | R |  |
| *Mouse IL-1β* | F | TGGACCTTCCAGGATGAGGACA  GTTCATCTCGGAGCCTGTAGTG |
|  | R |  |
| *Mouse TNF-α* | F | GGTGCCTATGTCTCAGCCTCTT  GCCATAGAACTGATGAGAGGGAG |
|  | R |  |
| *Mouse GAPDH* | F | CATCACTGCCACCCAGAAGACTG  ATGCCAGTGAGCTTCCCGTTCAG |
|  | R |  |

Abbreviations: F, forward; R, reverse; GAPDH, glyceraldehyde-3-phosphate dehydrogenase; IL, interleukin; TNF, tumour necrosis factor


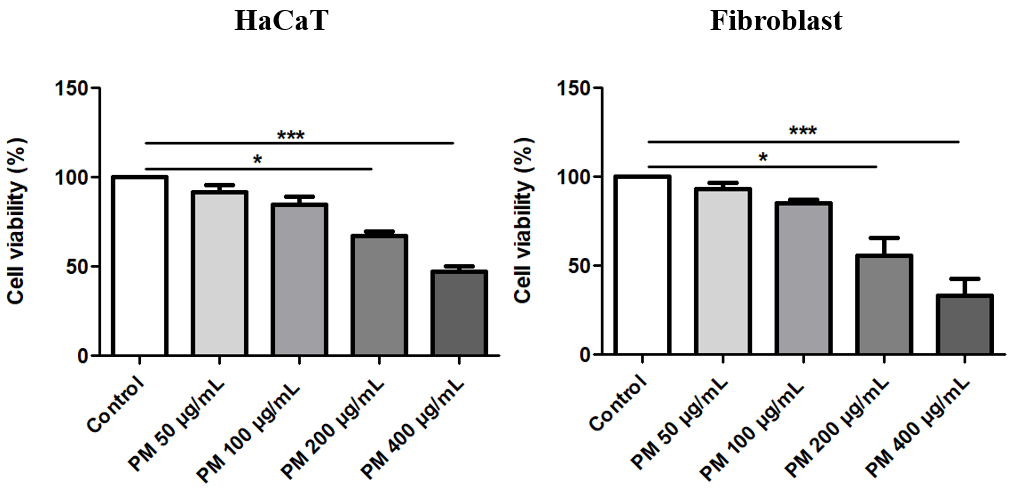


**Supplementary Figure S1. Cell proliferation was assessed using the CCK-8 assay.** The viability of HaCaT cells and fibroblasts decreased in a concentration-dependent manner following PM2.5 exposure. Five independent biological replicates were analyzed. The data represent the mean ± SD. (*p < 0.05, ***p<0.001)


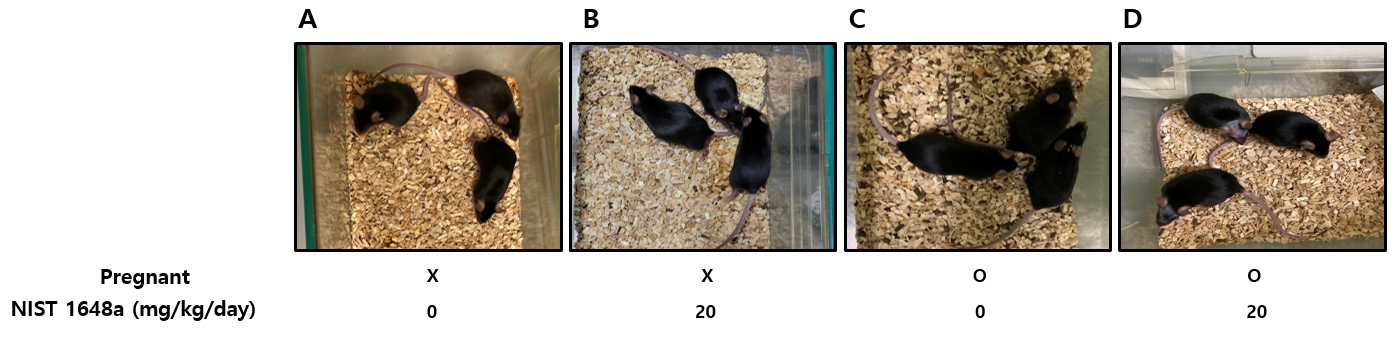


**Supplementary Figure S2. Representative images of control or PM 2.5 exposure in non-pregnant and postpartum mice.** (A) Non-pregnant control (B) Non- pregnant exposed to PM2.5 (C) Postpartum control (D) Postpartum exposed to PM2.5.


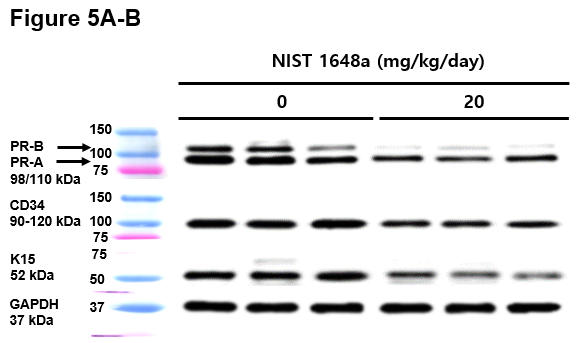

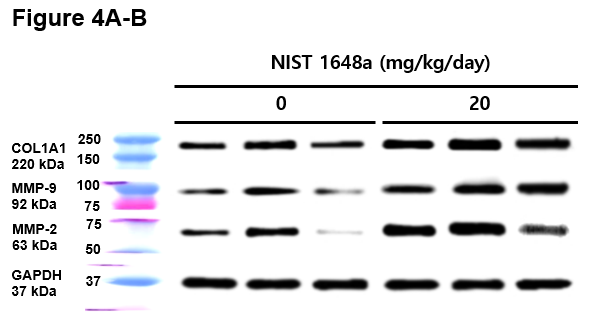

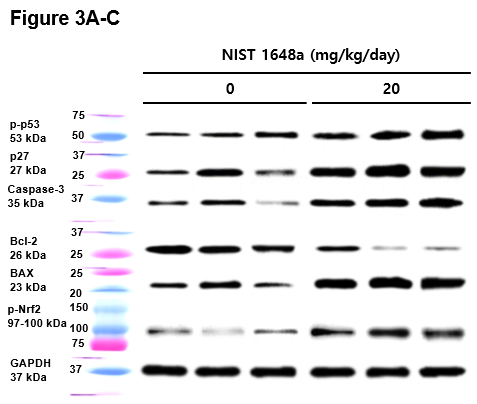

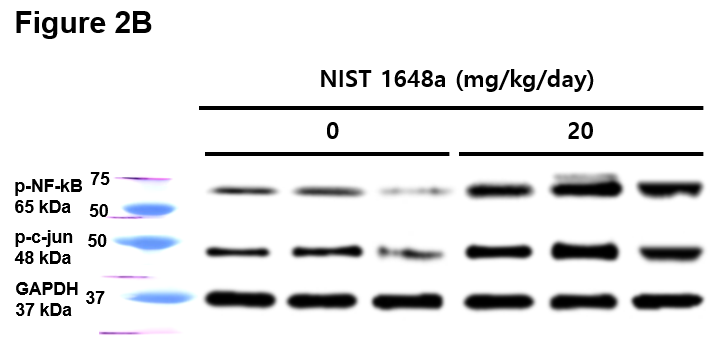


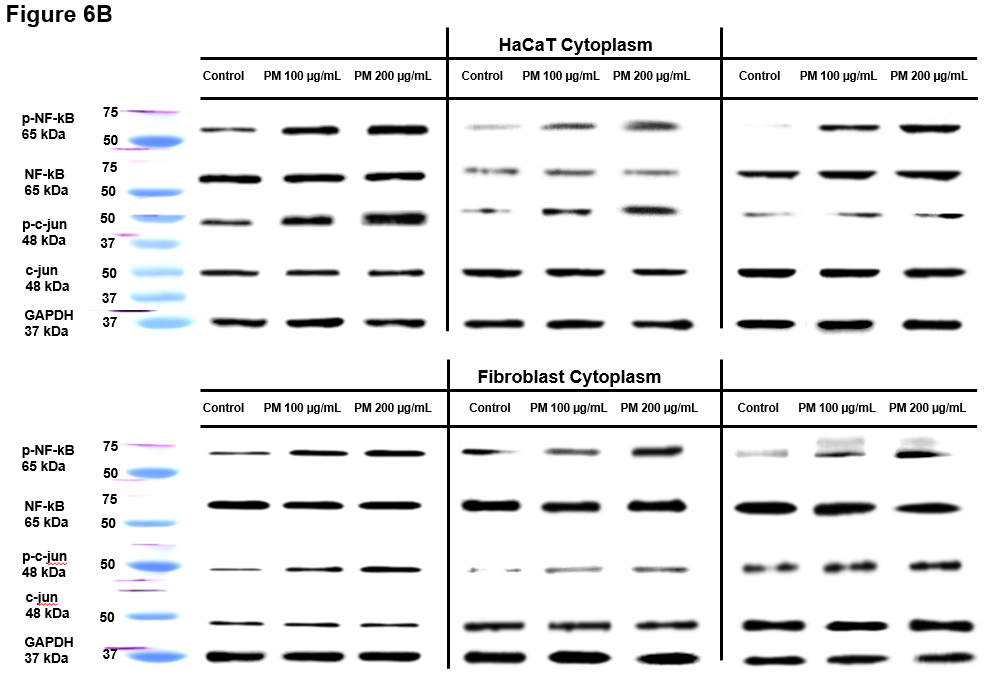


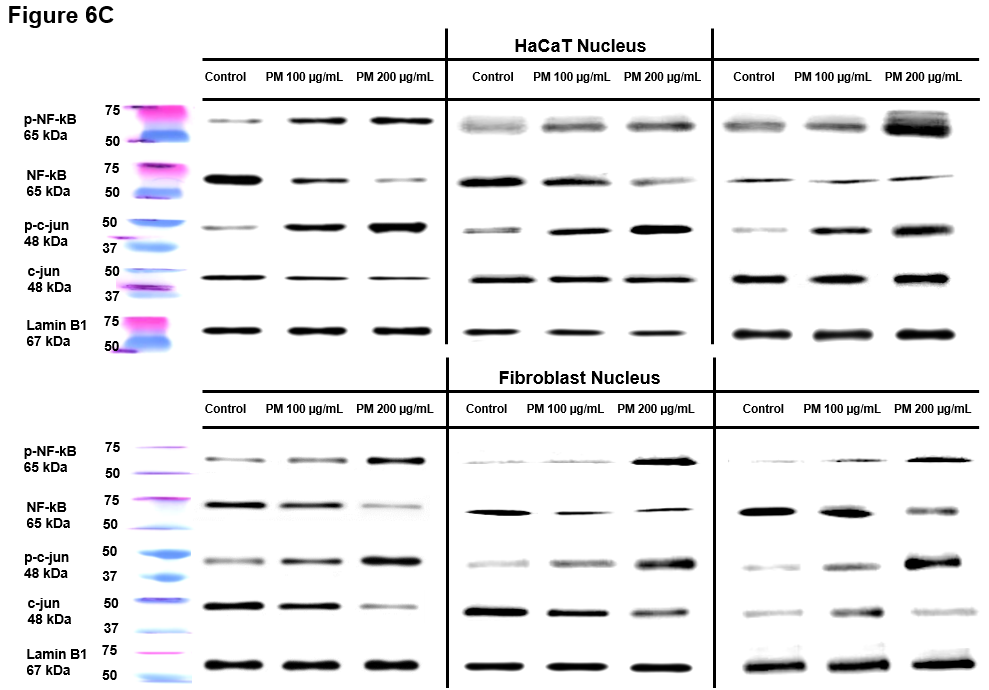


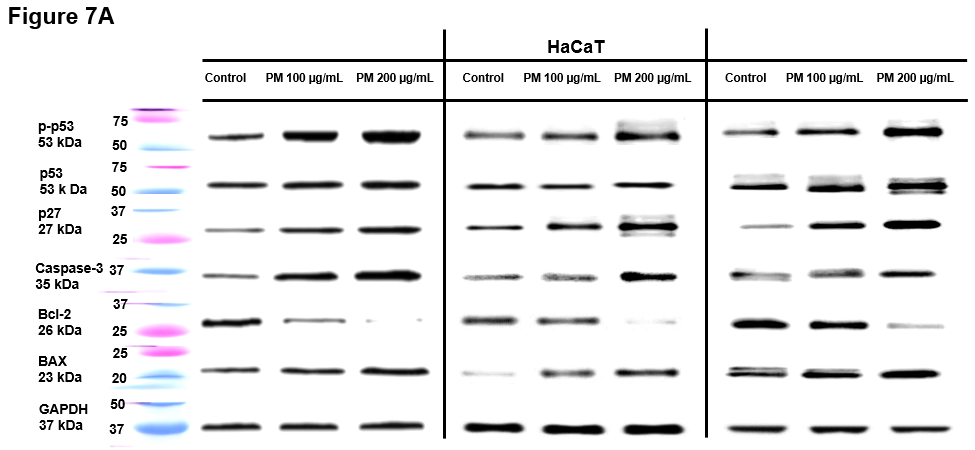


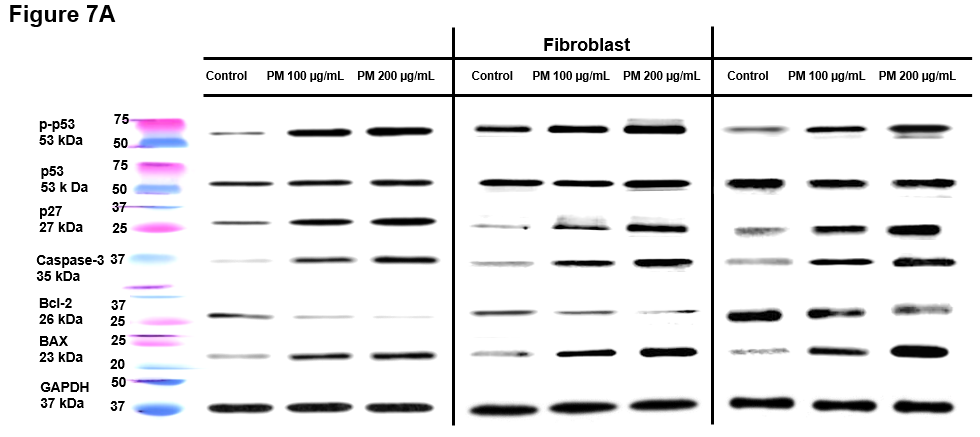


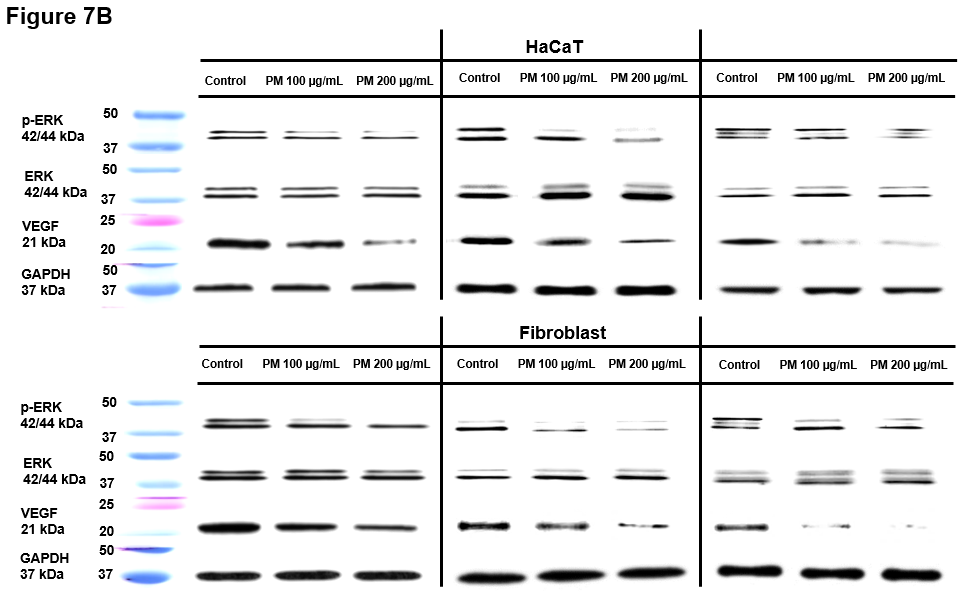


**Supplementary Figure S3. Original western blot images corresponding to the main figures.** Images show original membrane regions with visible molecular weight markers. Three biological replicates are shown.
